# Social media influence on eating disorder: a pilot study

**DOI:** 10.1192/j.eurpsy.2025.494

**Published:** 2025-08-26

**Authors:** B. Morigine, G. Spennato, E. Scopetta, J. S. Napoli, A. Napoli, H. Lamberti, O. Scicolone, F. Micanti

**Affiliations:** 1 UOC Psichiatria, Federico II, Napoli, Italy

## Abstract

**Introduction:**

Eating disorders are a group of mental illnesses determining general health consequences. Several studies suggest that social media influence body image concerns, considered the core of eating disorder pathology. Thin idealization has become an increasing cultural focus, leading young people to pursue body image as a symbol of success.

**Objectives:**

Aim of this study is to assess the quality and quantity of Social Network (SN) use and their influence on body uneasiness in individuals with Disordered Eating Behaviours (DEB) or Eating Disorders(ED)

**Methods:**

69 individuals suffering from Disordered Eating Behaviours (Grazing, Sweeteating, Food Addiction) or Eating Disorders (Night Eating Syndrome, Anorexia nervosa, Bulimia nervosa or Binge eating disorder) were enrolled. Mean age was 34 (SD ±11,33), mean BMI 28,68 (SD ±12,23). 93% of individuals were females. 20,29% (14) of the sample suffered from ED and 79,71% (55) from DEB. A social network self-administered questionnaire was used to investigate which SN was most used and what contents were most shown in users’ feed; domains investigated by the questionnaire are reported in fig. 1

**Results:**

Questionnaire analysis allowed to investigate individuals preferences about social media use and the kind of contents they consumed the most. The data showed the most frequently used social network are Facebook (42%), Instagram (33%) and Tiktok (22%). Healthy and/or fit contents were frequently displayed on SN feed 57.9% (40), but only 26,08% (18) of users were influenced in their lifestyle and food habits. 39,13% (27) of the individuals also felt like this kind of contents increased their sense of body uneasiness.

The study emphasize that 42,85% of the individuals diagnosed with an ED answered that SN content influenced their lifestyle and 71,42% that it increased their feeling of body uneasiness. In individuals with DEB only 21,8% were influenced in their lifestyle and 30,9% describe a worsening of their body uneasiness, as is shown in Tab.1.

**Tab 1:** 

	Lifestyle and food habits	Body uneasiness	*N of individuals*
**ED**	42,85%	71,42%	14
**DEB**	21,8%	30,9%	55
**ED+DEB**	26,08%	39,13%	69

**Image 1:**

**Figure:**
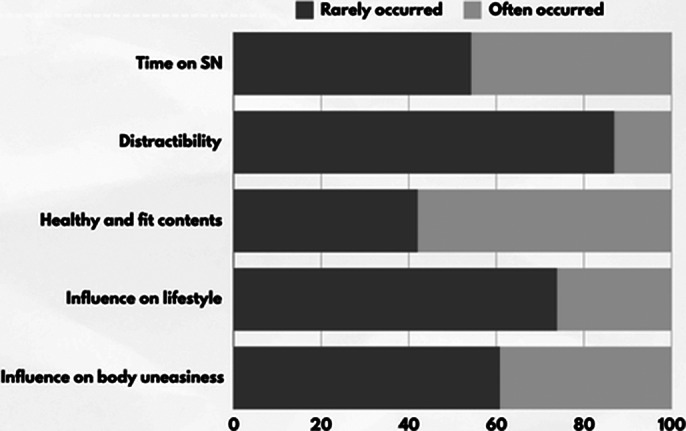

**Conclusions:**

Social media contents about health, diet and sport impact on individuals body image, diet and body uneasiness, mostly in individuals diagnosed with ED. This evidence stresses the importance to take into account every component of ED and DEB for a global approach to individuals including an assessment on interests, hobbies and use of social media.

**Disclosure of Interest:**

None Declared

